# Challenging Giant Insular Gliomas With Brain Mapping: Evaluation of Neurosurgical, Neurological, Neuropsychological, and Quality of Life Results in a Large Mono-Institutional Series

**DOI:** 10.3389/fonc.2021.629166

**Published:** 2021-03-22

**Authors:** Marco Rossi, Lorenzo Gay, Marco Conti Nibali, Tommaso Sciortino, Federico Ambrogi, Antonella Leonetti, Guglielmo Puglisi, Henrietta Howells, Paola Zito, Federico Villa, Gjulio Ciroi, Marco Riva, Lorenzo Bello

**Affiliations:** ^1^ Neurosurgical Oncological Unit, Department of Oncology and Hemato-Oncology, Università Degli Studi di Milano, Milano, Italy; ^2^ Laboratory of Medical Statistics, Biometry, and Epidemiology “G.A. Maccararo,” Department of Clinical Sciences and Community Health, Università degli Studi di Milano, Milano, Italy; ^3^ Laboratory of Motor Control, Department of Medical Biotechnology and Translational Medicine, Università degli Studi di Milano, Laboratorio Interdisciplinare di Tecnologie Avanzate (LITA), Milano, Italy; ^4^ Department of Anesthesia and Intensive Care, Humanitas Research Hospital, IRCCS, Milano, Italy

**Keywords:** extent of resection, glioma, insula, neuropsychological evaluation, quality of life, brain mapping, awake surgery

## Abstract

**Objective:**

Giant insular tumors are commonly not amenable to complete resection and are associated with a high postoperative morbidity rate. Transcortical approach and brain mapping techniques allow to identify peri-insular functional networks and, with neurophysiological monitoring, to reduce vascular-associated insults. Cognitive functions to be mapped are still under debate, and the analysis of the functional risk of surgery is currently limited to neurological examination. This work aimed to investigate the neurosurgical outcome (extent of resection, EOR) and functional impact of giant insular gliomas resection, focusing on neuropsychological and Quality of Life (QoL) outcomes.

**Methods:**

In our retrospective analysis, we included all patients admitted in a five-year period with a radiological diagnosis of giant insular glioma. A transcortical approach was adopted in all cases. Resections were pursued up to functional boundaries defined intraoperatively by brain mapping techniques. We examined clinical, radiological, and intra-operative factors possibly affecting EOR and postoperative neurological, neuropsychological, and Quality of Life (QoL) outcomes.

**Results:**

We finally enrolled 95 patients in the analysis. Mean EOR was 92.3%. A Gross Total Resection (GTR) was obtained in 70 cases (73.7%). Five patients reported permanent morbidity (aphasia in 3, 3.2%, and superior quadrantanopia in 2, 2.1%). Suboptimal EOR associated with poor seizures control postoperatively. Extensive intraoperative mapping (inclusive of cognitive, visual, and haptic functions) decreased long-term neurological, neuropsychological, and QoL morbidity and increased EOR. Tumor infiltration of deep perforators (vessels arising either medial to lenticulostriate arteries through the anterior perforated substance or from the anterior choroidal artery) associated with a higher chance of postoperative ischemia in consonant areas, with the persistence of new-onset motor deficits 1-month post-op, and with minor EOR. Ischemic insults in eloquent sites represented the leading factor for long-term neurological and neuropsychological morbidity.

**Conclusion:**

In giant insular gliomas, the use of a transcortical approach with extensive brain mapping under awake anesthesia ensures broad insular exposure and extension of the surgical resection preserving patients’ functional integrity. The relation between tumor mass and deep perforators predicts perioperative ischemic insults, the most relevant risk factor for long-term and permanent postoperative morbidity.

## Introduction

Resection of insular gliomas always represented a challenge for neurosurgeons because of the complex functional involvement of the insular lobe and opercula and the intricate vascularization of the area. Various surgical approaches have been proposed, with different rates of postoperative morbidity and extent of resection (EOR) (1, 2). The insula has recently been divided into four quadrants on a sagittal view based on bisection of the lobe on a horizontal plane along the Sylvian fissure and a perpendicular plane along the foramen of Monro (3, 4); insular tumors are therefore categorized according to prevalent tumor location. Each insular zone presents a different degree of surgical accessibility and functional and vascular involvement; generally, tumors belonging to zones I and IV (anterosuperior and anteroinferior quadrants, respectively) are those with the higher chance of complete resection, while tumors localized in zones II and III (posterosuperior and posteroinferior quadrants, respectively) present major surgical difficulties, a minor extent of resection, and a higher rate of permanent morbidity. Insular gliomas occupying all four zones are termed “giant” and are the most demanding: they are less amenable of complete resection and, in the most extensive series, associated with the highest rate of neurological morbidity due to delicate surgical access to this area, frequent larger tumor volume at diagnosis, and involvement of many surrounding functional networks and vascular structures (3–6). An entanglement of eloquent subcortical tracts encloses the insular lobe. The inferior fronto-occipital fasciculus (IFOF) and the uncinate fasciculus (UF) run inferiorly and antero-inferiorly to the insula (6). The transopercular corridor to get to the insula is delimited, in the frontal lobe, by the superior longitudinal fascicle III (SLF III) and the arcuate fascicle (AF) long segment superiorly, the AF posterior segment postero-superiorly; in the temporal lobe, by the AF terminations in the posterior portion of the superior temporal gyrus, the IFOF and optic radiations superiorly and posteriorly, and the inferior longitudinal fascicle (ILF) inferiorly and posteriorly. Main vascular structures to deal with the insular lobe include: the middle cerebral artery (MCA) and its opercular branches anteriorly and laterally; the lenticulostriate arteries (LSA complex); and other deep perforators arising from the anterior choroidal artery (AChA).

Insular gliomas are often characterized by cognitive impairment and seizures hard-to-control (7–9). Recent studies highlighted the role of surgical resection in insular gliomas and its influence on progression free-survival, on postponing malignant transformation, and on seizure control (5, 7, 10–14). Although these data stressed the need to improve resection also in the giant subtype, the preservation at the same time of patients’ functional integrity is a challenge. When a transcortical approach is adopted, the number and type of functions to be mapped and surgical corridors are still under debate. Furthermore, the functional risk of surgery has been traditionally evaluated merely by neurological examination, with sporadic or any data about the impact on neuropsychological profile and Quality of Life (QoL) of subjects. In this work, we analyzed the impact on Extent of Resection (EOR) and functional (neurological, neuro-psychological, and QoL evaluations) results of surgical resection in 95 giant insular gliomas, operated adopting a transcortical approach. We aimed to investigate factors influencing the achievement of an extended resection and patient functional preservation, focusing the analysis on the impact on the neuropsychological profile and QoL.

## Materials and Methods

### Patients

Patients admitted from May 2013 to November 2018 harboring a radiological diagnosis of giant insular gliomas (*i.e.*, a mass lesion involving all four insular zones) and candidate for resection were included. All patients gave written informed consent to the surgical procedure, covered by Ethical-Committee IRB-1299 Humanitas Research Hospital.

### Imaging and EOR

MR images were performed on a Philips Intera 3.0T pre-operatively, within 48 h of and 1 month after surgery. The protocol for lesion morphological and dimensional assessment and EOR estimation included: a) axial three-dimensional-FLAIR; b) post-gadolinium three-dimensional-T1-weighted fast-field-echo (MPRAGE); c) DWI and ADC imaging.

To calculate tumor volume (in cm^3^) and EOR, volumetric scan images were analyzed with semi-automatic segmentation using iPlanCranial software (BrainLab, Germany) by four blinded investigators (MRo, LG, MCN, TS); FLAIR hyperintense or T1-weighted gadolinium-enhanced signal abnormalities were included in the lesion load for non-contrast enhancing lesions or high-grade gliomas, respectively. The EOR corresponded to the percentage of preoperative tumor volume resected: (preoperative volume − postoperative volume)/preoperative volume (15). It was classified as: Gross Total Resection (GTR, EOR = 100%); Subtotal Resection (EOR <100–90%); Partial Resection (EOR <90%). In the analysis, Subtotal and Partial Resections were considered together.

Individual vascular anatomy and vessels relation to tumor mass were evaluated on preoperative post-gadolinium three-dimensional-T1-weighted images, ischemia on immediate postoperative DWI; the amount of DWI signal was considered significant when more than a small punctum or small rim, i.e., focal areas >2 ml in volume around the resection cavity or in other subcortical areas were detected. The location of the DWI abnormalities and eloquent subcortical sites involvement were evaluated; a subcortical site was considered eloquent when located within the course of tracts mediating language, cognitive, motor, praxis, or visual functions.

### Surgical Procedures

**Figure 1:**
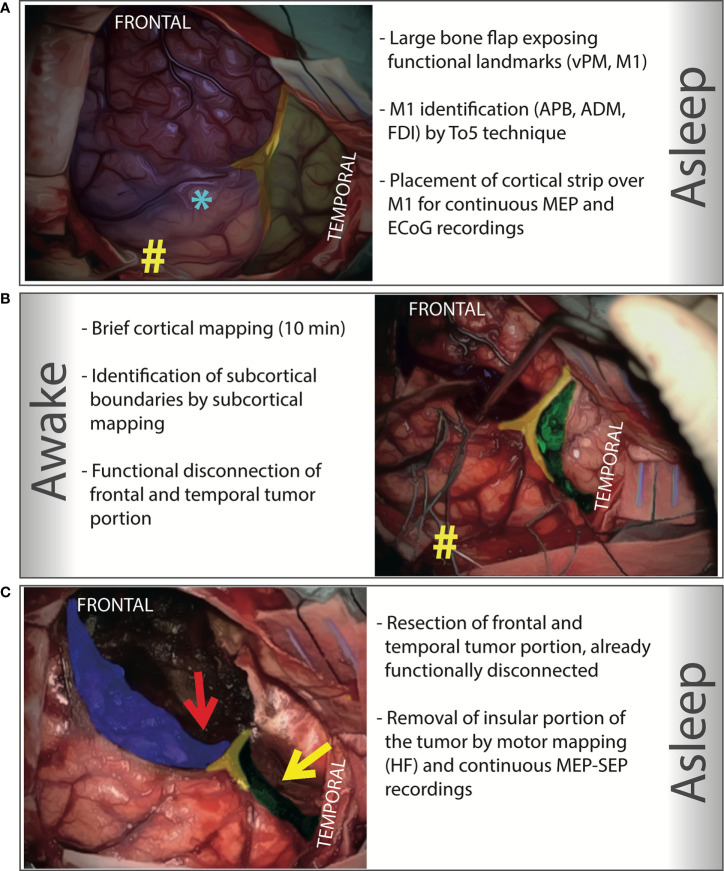
Step-by-Step illustration of an asleep-awake-asleep transcortical approach to a right giant insular low-grade glioma. Intraoperative pictures have been colored with different inks to highlight the key elements of each step. **(A)** A large bone flap exposing the functional cortical landmark (M1 and vPM, marked with # and *, respectively) is designed and performed under general anesthesia. The motor cortex is identified (by HF stimulation, Train of Five, ISI 3, delivered by a monopolar probe), looking for hand muscle responses (APB, ADM, FDI) at cortical motor threshold (the lowest current intensity which produced the lowest MEP response); a cortical strip is placed over this area to obtain a continuous MEP monitoring; the same strip is also used to record ECoG. (in *blue* is colored the frontal lobe, in *green* the temporal lobe, and in *yellow* the Sylvian fissure). **(B)** The awake phase consists of a cortical and a subcortical step. In the cortical step, which last approximately 10 minutes, by cortical mapping (LF delivered by a bipolar probe) functional cortical sites are located over the frontal and the temporal lobe, and negative cortical sites identified to design the safe cortical entry zone in both frontal and the temporal lobe; the corticectomy is then performed at the posterior border of both lobes. In the subcortical step (20-30 minutes on average), with the aid of subcortical mapping, the functional limits in the frontal and temporal lobes are identified. Limits are immediately sought at the periphery, starting from the corticectomies. In this way, a functional disconnection of the frontal and temporal portion of the tumor is progressively obtained. Resection is precisely achieved according to functional limits, always identified, and representing the resection limits in the frontal and temporal lobe. Functional subcortical margins are marked and secured with patties (in the figure, the subcortical functional margin in the frontal lobe is marked with patties; a neuro-navigation probe is also placed inside its inferior portion; *green*: subcortical functional margin at temporal level; *yellow*: the Sylvian fissure; #: the tip of the cortical strip). **(C)** The last phase is performed under general anesthesia. The tumor frontal and temporal portions are resected according to the functional boundaries defined during the awake phase. This maneuver highlights the tumor insular portion, either through the frontal (*red arrow*) or temporal (*yellow arrow*) window. Afterwards, the insular portion of the tumor is removed with the aid of HF motor mapping and continuous MEP-SEP monitoring. (In *blue* highlighted the subcortical functional margin at frontal level; in *green* the subcortical functional margin at temporal level; in *yellow* the Sylvian fissure).

All surgical resections were pursued up to functional boundaries (motor, language, haptic, visual, and cognitive), aiming to achieve a Gross Total Resection (GTR) whenever possible, without any patient or tumor *a priori* selection. Surgery was performed under either asleep-awake-asleep anesthesia or general anesthesia, with the aid of Mapping and Monitoring technique (16). Mapping includes Low- and High-Frequency (LF, HF) direct electrical stimulation (DES) (17). Monitoring includes EEG, electrocorticography (ECOG), motor evoked potentials (MEPs), somatosensory evoked potentials (SEPs) recordings. Patients were positioned supine, with the shoulder elevated of 30° and the head tilted of 30° toward the tumor opposite side. A large fronto-temporal flap was designed. The craniotomy, performed under general anesthesia, exposed the tumor area and an amount of surrounding tissue, including functional cortical landmarks, *i.e.*, the ventral pre-motor area (vPM) and the motor cortex (M1) (**Figure 1**). Cortical mapping was initially performed with HF stimulation (Train of Five, ISI 3) delivered by a monopolar probe to identify M1, specifically the hand knob, looking for the cortical motor threshold (cMT, *i.e.* the lowest current intensity which produced the lowest MEP response) for three hand muscles (*Abductor Pollicis Brevis*, *Extensor Digitorum Comunis*, *Abductor Digiti Minimi*) (18). The cortical strip was then placed over this area for continuous ECoG and MEP recordings. In asleep–awake–asleep procedures, the working current interfering with counting was then established over vPM. The awake phase consisted of both a cortical and a subcortical step. In the cortical step, LF stimulation delivered with a bipolar probe mapped functional cortical sites; negative cortical sites were identified to design the safe cortical entry zone for corticectomy, over the frontal and temporal lobes. In the subcortical step, with the aid of subcortical mapping by LF stimulation, functional limits were sought at the periphery of the opercular windows outlined, starting from the corticectomies. Positive sites (discrete regions where DES produced transient functional disturbance) where marked and secured with patties, representing the surgical resection limits. In this way, a functional disconnection of tumor frontal and temporal portion was progressively obtained. The neuro-psychological tasks performed were tailored to the region of stimulation. Language mapping was performed with naming and semantic association tasks. Praxis mapping using the Hand Manipulation Task (hMT) (19). Cognitive mapping (attentive and executive functions and memory) was eventually deployed (20). To preserve visual function during temporal lobe resection the Visual field Task was utilized (21). The last phase, proper tumor removal, was performed under general anesthesia also in asleep–awake–asleep procedures. Frontal and temporal portions of the tumor were resected according to the functional boundaries previously defined. By this maneuver, exposure of the insular lobe was achieved either through the frontal or the temporal window. Afterward, the insular portion of the tumor was removed with the aid of HF motor mapping by monopolar probe, up to a 2–3 mA sMT was eventually reached, and continuous MEP, SSEP, and EEG monitoring. Dissection was discontinued when MEP recordings showed either fluctuation of at least 2 out of the 3 recorded hand muscles or 50% amplitude reduction. Irrigation with saline and increase in arterial pressure in these circumstances were applied to let MEP recover.

According to the different surgical strategies adopted, the series was categorized in two groups: 1) 28 patients treated before 2015, among which less than 60% of the surgeries (all dominant hemisphere lesions) were performed under awake settings, mostly with mapping limited to motor function and language; 2) 67 patients treated after 2015, among which all patients, if clinically feasible and irrespective of hemisphere involved, were operated under asleep-awake-asleep regimen (>75%) with an extensive mapping involving also cognitive, haptic, and visual tasks.

### Morbidity, Neuropsychological Profile, and QoL Evaluation

Examinations were performed before, on the 5th day, 1–3 months, and 1-year after surgery. Morbidity was assessed by evaluating postoperative new-onset neurological deficits and the occurrence of intraoperative complications (e.g., intra-cavitary bleeding or ischemia). Intraoperative mortality was also reported.

The neuropsychological profile was evaluated with the *Milano-Bicocca-battery* (22), assessing five neurocognitive domains: language, memory, visuospatial abilities and praxis, attentive and executive functions, and fluid-intelligence. Each test score was corrected for patient age and educational level to obtain a normalized value and converted to an equivalent score on a scale of five values from 0 to 4 (0 = pathological, 1–4 = different degrees of normal performance) (23). The division of the corrected scores into five different regions, each corresponding to an equivalent score, was based on the distribution of the scores observed in a control population; the first region (equivalent score = 0) corresponds to the worst 5th percentile results observed in the control population, while the region on the opposite end (equivalent score = 4) corresponds to the corrected scores in the control population between the median and the maximum score. The other three regions (1, 2, and 3) are defined between these two extreme regions so that the ranges of the corrected scores observed are evenly spaced between the normality reference threshold and median. This conversion in equivalent scores allowed the comparison of test scores among different subjects. For the analysis, pathological versus normal test scores were considered.

Quality of Life was assessed as global performance status, ability and time to return to work, and by the Short Form (36) Health Survey questionnaire (SF-36) and Hospital Anxiety and Depression Scale (HADS) (24). To provide a single score in the SF-36, we assumed the “General Health scale” (GH scale) score as an indicator of the QoL. The GH scale scores of patients under analysis were compared to the average score recorded in the Italian general control population; patients with a score one standard deviation below the average score of the Italian control group were classified as patients with a low perception of their QoL (25). The HADS Italian version cut-off point of 10 was adopted to identify patients with emotional disorders (26).

### Factors Related to EOR and Functional Outcome

Factors possibly associated with EOR and functional outcome included in the analysis are illustrated in **Table 1**.

**Table 1:** Clinical, imaging, pathological, and surgical features.

Category			GTR	Partial/Subtotal	*p-value*	New-onset neurological deficit at 1 month	*p-value*	New-onset NPS deficit at 3 months	*p-value*
**No. of patients, n (%)**		95 (100)	70 (73.7)	25 (26.3)		24 (25.2)		33 (34.7)	
*Pre-operative factors*									
**Age, years**	Range	19–66.8	21.5–66.8	19–60.3	*0.565*	22.4–60.3	*0.506*	25.1–66.8	*0.705*
	Median	40.16	40.6	37.8		35.8		39.1	
	Mean	40.89	41.3	39.6		39.5		40.3	
**Sex, n (%)**	Males	59 (62)	40 (67.8)	19 (32.2)	*0.149*	13 (22)	*0.466*	24 (40.7)	*0.173*
	Females	36 (38)	30 (83.3)	6 (16.7)		11 (30.5)		9 (25)	
**Previous surgery, n (%)**	No	50 (52.6)	37 (74)	13 (26)	*1.000*	15 (30)	*0.346*	19 (38)	*0.660*
	Previous surgery	45 (47.4)	33 (73.3)	12 (26.7)		9 (20)		14 (31.1)	
	*Biopsy*	27 (28.5)							
	*Surgery in other lobes*	18 (18.9)							
**Clinical history, n (%)**	>6 months	43 (45.3)	31 (72.1)	12 (27.9)	*0.817*	8 (18.6)	*0.251*	14 (32.6)	*0.827*
	<6 months	52 (54.7)	39 (75)	13 (25)		16 (30.8)		19 (36.5)	
**Neurological deficit, n (%)**		28 (29.5)	19 (67.8)	9 (32.2)	*0.448*				
	Language	16 (16.8)	11 (68.7)	5 (32.2)		–	*-*	–	*-*
	Motor	11 (11.5)	6 (54.5)	5 (45.4)					
	Visual	3 (3.15)	2 (66.7)	1 (33.3)					
**Seizures, n (%)**		70 (73.7)	49 (70)	21 (30)	*0.198*	17 (24.3)	*0.790*	28 (40)	*0.129*
	Focal	42 (60)	28 (66.7)	14 (33.3)		10 (23.8)		19 (45.2)	
	Generalized	28 (40)	21 (75)	7 (25)		7 (25)		9 (32.1)	
**Seizures control, n (%)**	Control	52 (54.7)	44 (84.6)	8 (15.4)	***0.010***	13 (25)	*1.000*	20 (38.5)	*0.507*
	No	43 (45.3)	26 (60.5)	17 (39.5)		11 (25.6)		13 (30.2)	
**Anti-epileptic drugs (AED) n., n (%)**	No AED	14 (14.7)	9 (64.3)	5 (35.7)	*0.539*	–	*-*	–	*-*
	1	50 (52.6)	39 (78)	11 (22)					
	>1	31 (32.7)	22 (70.9)	9 (29.1)					
**Handedness, n (%)**	Left	3 (3.15)	2 (66.7)	1 (33.3)	*0.605*	–	*-*	–	*-*
	Right	92 (96.85)	68 (73.9)	24 (26.1)					
*Pre-operative MRI features*									
**Side, n (%)**	Left	60 (63.15)	43 (71.7)	17 (28.3)	*0.634*	17 (28.3)	*0.466*	24 (0)	*0.257*
	Right	35 (36.85)	27 (77.1)	8 (22.9)		7 (20)		9 (25.7)	
**Location, n (%)**	Pure insular	14 (14.7)	11 (78.6)	3 (21.4)	*0.754*	2 (14.3)	*0.506*	4 (28.6)	*0.759*
	Opercular extension	81 (85.3)	59 (72.8)	22 (27.2)		22 (27.2)		29 (35.8)	
**MRI borders, n (%)**	Diffuse	60 (63.1)	44 (73.3)	16 (26.4)	*1.000*	18 (30)	*0.330*	18 (30)	*0.262*
	Compact	35 (37.9)	26 (74.3)	9 (25.7)		6 (17.2)		15 (42.8)	
**Pre-op volume in mL**	Range	17.1–144.1	17.1–144.1	28.2–128.9	*0.472*	20.3–116.1	*0.368*	18.9–115.5	*0.721*
	Median	71.12	77.43	74.98		69.7		71.7	
	Mean	76.74	73.13	76.84		72.2		75.2	
**Relation of LSA, n (%)**	LSA mesial to tumor	49 (51.6)	36 (73.5)	13 (26.5)	*1.000*	15 (30.6)	*0.158*	15 (30.6)	*0.510*
	LSA within the tumor	46 (48.4)	34 (73.9)	12 (26.1)		9 (19.5)		18 (39.1)	
**Relation of deep perforators, n (%)**	tumor invasion	32 (33.7)	18 (56.2)	14 (43.8)	***0.012***	6 (18.7)	*0.216*	13 (40.6)	*0.355*
	no relation	63 (66.3)	52 (82.5)	11 (17.5)		18 (28.5)		20 (31.7)	
**Relation of opercular branches, n (%)**	All involved	58 (61.1)	40 (68.9)	18 (31.1)	*0.236*	11 (18.9)	*0.287*	22 (37.9)	*0.376*
	Not all involved	37 (38.9)	30 (81)	7 (19)		13 (35.1)		11 (29.7)	
*Tumors factors*									
**Histology, n (%)**	Oligodendroglioma	34 (35.8)	26 (76.5)	8 (23.5)	*0.318*	8 (23.5)	*0.911*	13 (38.2)	*0.101*
	Astrocytoma	42 (44.2)	28 (66.7)	14 (33.3)		11 (26.2)		18 (42.8)	
	Glioblastoma	19 (20)	16 (84.2)	3 (15.8)		5 (29.4)		2 (10.5)	
**Histological Grade, n (%)**	Low	69 (73.7)	48 (69.6)	21 (30.4)	*0.193*	18 (26.1)	*0.786*	28 (40.6)	*0.129*
	High	26 (26.3)	22 (84.6)	4 (15.4)		5 (19.2)		5/22 (19.2)	
**IDH1, n (%)**	Mutated	77 (81.1)	57 (74)	20 (26)	*1.000*	21 (27.7)	*1.000*	28 (36.4)	*0.756*
	Wild type	18 (18.9)	13 (72.2)	5 (27.8)		5 (27.7)			
*Intraoperative Factors*									
**Anesthesia, n (%)**	Awake	70 (73.6)	52 (74.3)	18 (25.7)	*0.791*	20 (28.6)	*0.287*	28 (40.3)	*0.293*
	Asleep	25 (26.4)	18 (72)	7 (28)		4 (16)		5 (20)	
**Mean duration of surgery (min.)**		280 ± 30	285 ± 22	278 ± 28	*–*	276 ± 25	*–*	282 ± 26	*–*
**Brain mapping, n (%)**	Limited	15 (21.5)	7 (46.7)	8 (53.3)	***0.010***	7 (46.7)	*0.201*	8 (53.3)	*0.192*
	Extended	55 (78.5)	45 (81.8)	10 (18.2)		17 (30.9)		19 (34.5)	
**Mapping time (min.)**		39 ± 11	40 ± 8	38 ± 10	*–*	39 ± 8	*–*	40 ± 6	*–*
**Severe MEP variations, n (%)**		4 (4.2)	3 (75)	1 (25)	*1.000*	*–*	*–*	–	*–*
**Reported patient’s fatigue, n (%)**		2 (2.1)	2 (100)	0	*0.539*	0	*–*	2 (9.1)	
**Operative mortality, n (%)**		0	0	0	*–*	0	*–*	0	*–*
*Post-operative factors*									
**Ischemia on post-op DWI, n (%)**		19 (20)	14 (21.9)	5 (21.7)	*0.620*	8 (42.1)	*0.380*	7 (36.8)	*0.466*
**EOR, n (%)**	GTR	70 (73.7)	*–*	*–*	*–*	20 (28.6)	*0.287*	22 (31.4)	*0.129*
	Subtotal/Partial resection	25 (26.3)				4 (16)		11 (44)	
**Seizures control, n (%)**	control	88 (92.6)	69 (98.5)	19 (76)	***0.001***	20 (22.7)	*0.366*	29 (32.9)	*0.205*
	no	7 (7.4)	1 (1.5)	6 (24)		4 (57.1)		4 (57.1)	

Clinical, imaging, pathological, and surgical features of the 95 patients with giant insular gliomas operated according to functional boundaries via transcortical approach and finally enrolled in our retrospective analysis. Numerosity and percentages are divided into subgroups by means of EOR, presence of neurological deficits of new-onset 1-month post-op, and presence of neuropsychological deficits of new-onset 3-months post-op. P-values refer to the association (chi-square and Fisher’s exact test) between factors analyzed and EOR subgroups, presence of new-onset neurological deficit 1-month post-op, and presence of new-onset neuropsychological deficit 3 months post-op. EOR is classified as gross-total-resection (GTR) versus Subtotal and Partial resections. Previous surgery displays whether patients received previous surgical treatment, performed at other institutions (needle or open biopsy, partial resection of opercula). Neurological deficits included: motor deficits (VII cranial nerve central deficit and/or mild contralateral limbs hyposthenia), visual deficits (superior quadrantanopia), and language deficits. Tumor location is indicated as pure insular versus opercular extension (frontal and temporal) of the lesion. Tumor borders refer to borders appearance on pre-operative volumetric MR and are classified as well-defined vs. irregular, using post-contrast imaging for contrast lesions or FLAIR images for those that were non-enhancing. Histology was classified according to the recent WHO 2016 Classification. IDH1-status was determined by immunohistochemistry and, in case of negative expression, by mutational analysis. ATRX and 1p19q codeletion were routinely performed (by immunohistochemistry and FISH, respectively) in all excised tumor tissues to support the exact histological diagnosis molecularly. “Intraoperative mapping was categorized as “limited” when it included motor and language mapping only, and “extended” when it also included haptic, visual, and cognitive testing. Patient fatigue indicates when the planned mapping was terminated earlier due to loss or reduction of appropriate patient collaboration. Postoperative seizure control was classified according to Engel Surgical Outcome Scale (seizures control = class I; no seizures control = class IV). In bold: P-values<0.05.

Peri-insular vascular structures, assessed on preoperative 3.0T volumetric T1-weighted post-Gadolinium scans, included: 1) lenticulostriate arteries (LSA complex) that branch off the M1 segment (less frequently M2) of the MCA; the relation of LSA to tumor mass was categorized as “*LSA mesial to the tumor*” when tumor mass got in proximity to or medially displayed the LSA and their origin without invading these vessels, and as “*LSA within the tumor*” when tumor mass encased the LSA. 2) Other deep perforators, branches that stem at the origin of the AChA from the internal carotid artery (ICA) and supply lateral part of the geniculate body, posterior two-thirds of the posterior limb of the internal capsule, most of the globus pallidus, the origin of the optic radiations, and the middle third of the cerebral peduncle; the relation of deep perforators to tumor mass was categorized as “*tumor invasion*” when these branches were encased by tumor volume, or as “*no relation*” when tumor mass medially displaced the deep perforators without engulfing them. 3) Opercular branches of the MCA (M2–M3 segments), specifically from the superior trunk, and running over the insular cortex: orbitofrontal, prefrontal, and precentral arteries; the relation of these structures was categorized as “*all involved*” by tumor when all the opercular branches were within the tumor mass, versus “*not all involved*” when at least one of the opercular arteries (anterior or precentral) was not involved by the tumor.

### Statistics

Chi-Square and Fisher’s exact test evaluated the association among factors. Continuous variables were categorized according to cutoffs reported in **Table 1**. The predictive value of the significant variables resulting from the univariate analysis was further assessed by multivariate regression analysis. Exact McNemar’s test was used for matched comparisons. Tests were performed with SPSS version 24.

## Results

### Patients

During the investigated period, out of 343 patients with insular gliomas consecutively admitted to our unit, 95 (27.7%) had a giant insular glioma (**Table 1**). Mean tumor volume was 76.74 ml. Most patients presented with seizures (73.7%); 43 out of 70 patients had poor seizure control, despite AEDs. Mild language and/or motor deficits were registered in 28 patients (29.5%).

### Surgical Procedure

A transcortical approach was always adopted. When awake mapping was performed (70 cases, 73.6%) the procedure was well tolerated (reported patient fatigue intraoperatively in 2.1%, absence of mapping abortion). Severe hand muscles MEP changes were observed in four cases; two of these recovered after surgical manipulation discontinuation, arterial pressure increase, and saline irrigation. The other two patients presented clinically relevant (contralateral hemiparesis) ischemic insult documented also on post-op DWI. All patients, at the 1-year evaluation, recovered to normal baseline neurologic examination.

### EOR and Associated Factors

Mean EOR was 92.3%, median 99%; a GTR was obtained in 70 patients (73.7%).

The achievement of GTR was not associated with any pre-operative clinical factor, except for seizure control in the pre-op that was significantly associated with a higher chance of GTR (**Table 1**). LSA relation to tumor mass, differently from current literature (27), was irrelevant. Instead, tumor involvement of the other deep perforators, opposed to medial displacement of these vessels by tumor mass, was associated with a minor chance of achieving a GTR (p = .012). EOR was associated with the type of brain mapping (p = .010): asleep–awake–asleep procedures with extended brain mapping with cognitive, haptic, and visual other than merely language testings, displayed a higher proportion of GTR.

### Morbidity and Neurological Outcome

Globally, the procedure was safe, and no intraoperative complications were recorded. No peri-operative mortality was documented.

In the pre-op, 28 patients presented with neurological disturbances (**Tables 1**, **2**). Immediately after surgery, half of patients developed new-onset postoperative deficits. The function most affected was language (32.6% of total, 75% of patients with dominant lesions). At one month, more than half of these patients recovered. At one year, language deficits persisted in three patients only (3.2% of the total, 6.3% of patients with dominant lesions) and affected speech-production in two and speech-production and comprehension in one patient. No motor deficits were reported. Two patients (2.1%) presented persistent superior quadrantanopia.

**Table 2:** Neurological and neuropsychological deficits.

Neurological deficits				
	Pre-op	Immediate post-op	1-month post-op	1-year post-op
**N. of patients, n (%)**	28 (29.5)	52 (54.7)	24 (25.2)	5 (5.2)
**Language**	16 (26.6)	31 (32.6)	20 (21.1)	3 (3.2)
		* 0(0)	* 0(0)	* 0(0)
**Motor**	11 (11.5)	26 (27.3)	7 (7.3)	0 (0)
		* 0(0)	* 3(27.2)	* 7(63.6)
**Visual**	3 (3.1)	3 (3.1)	3 (3.1)	2 (2.1)
		* 0(0)	* 0(0)	* 0(0)
**Praxis**	3 (3.1)	0 (0)	0 (0)	0 (0)
		* 0(0)	* 3(100)	* 3(100)
***Neuropsychological deficits***				
	**Pre-op**	**Immediate post-op**	**3 months post-op**	**1-year post-op**
**N. of patients, n (%)**	48 (50.5)	53 (55.8)	33 (34.7)	16 (16.8)
**Language**	20 (33.3)	34 (35.8)	16 (16.8)	6 (6.3)
		* 0(0)	* 0(0)	* 5(25)
**Memory**	46 (48.4)	17 (17.9)	7 (7.4)	4 (4.2)
		* 0(0)	* 0(0)	* 24(52.2)
**Visuospatial & praxial abilities**	10 (10.5)	22 (23.2)	12 (12.6)	4 (4.2)
		* 0(0)	* 0(0)	* 7(70)
**Attention and executive functions**	8 (8.4)	33 (34.7)	13 (13.7)	6 (6.3)
		* 0(0)	* 0(0)	* 6(75)
**Fluid intelligence**	5 (5.2)	6 (6.3)	3 (3.2)	1 (1.1)
		* 0(0)	* 0(0)	* 5(60)

The table reports the numerosity and percentages of neurological and neuropsychological deficits for the patients analyzed. Immediate post-op neurological deficits were evaluated at 5 days after surgery. Neuropsychological tests were performed preoperatively, 5 days post-op, at 1 and 3 months after surgery, and 1 year after. The score recorded at 3 months are presented. The Milano-Bicocca battery of tests was used in all patients. In the postoperative time points, are reported the numerosity of patients with new-onset pathological scores with respect to pre-op in at least one test of the neuropsychological domains indicated.

*Indicates the cumulative numerosity (and percentage) of patients with preoperative pathological scores/evaluations that improved to “normal” neuropsychological scores and neurological examination at the postoperative evaluation.

Forty-three patients had no or poor seizure control before surgery, despite AEDs. After surgery, poor seizure control was observed in seven patients only (Engel Outcome Scale class IVA and IVB) (Exact McNemar’s test, pre-op versus post-op, p = .000). Thirty-eight of 43 (88.4%) patients with no preoperative seizure control were seizure-free 1-year after surgery (Engel Outcome Scale class I); no increase in AEDs dose-number was reported. EOR was associated with seizure control (p = .001); six patients (24%) with subtotal/partial resection experienced poor seizure control compared to only one patient (1.5%) of those with GTR (**Table 1**).

### Neuropsychological Profile

Pre-operative neuropsychological scores were more affected than neurological performance (**Table 2**). Forty-eight patients (50.5%) presented pathological scores in at least one of the domains analyzed, most in memory (48.4%) and language (33.3%). Five days after surgery, most patients declined in at least one domain. Substantial recovery resulted at 3-month evaluation in all domains, but language and attentive and executive functions; language presented the slowest recovery rate. At 1-year evaluation, however, only six patients (6.3%) presented new-onset deficits in language, all in production (naming and/or verbal-fluency), three also in comprehension.

### QoL

QoL was evaluated considering the global performance status and ability and time to return to work. Seventy-four patients returned to work at 3 months, 92 at 6 months. Only 72 patients maintained the same working level. QoL evaluation (SF-36 and HADS scores) exhibited the same neuropsychological profile trend (**Table 3**).

**Table 3:** Quality of Life evaluation.

Quality of Life					
HADS (n. 95)					
**Grade**	**Pre-op, %**	**1-month post-op, %**	**3 months post-op, %**	**6 months post-op, %**	**1-year post-op, %**
LGG	35	53	45	36	28
HGG	38	53	65	72	65
**SF-36 (n. 95)**					
**Grade**	**Pre-op, %**	**1-month post-op, %**	**3 months post-op, %**	**6 months post-op, %**	**1-year post-op, %**
LGG	40	70	57	45	31
HGG	60	80	70	73	40

Quality of life: HADs and SF-36 score. The percentage of patients with pathological scores at the different time point of examination is reported. Scores are reported separately for patients diagnosed with a Lower-grade Gliomas (LGG, 69 patients) and with High-grade (IDH-1wild-type, HGG, 26 patients) gliomas. In the HGG group, scores are affected by adjuvant therapies; no recurrences were recorded.

### Factors Associated With Outcome

Neurological and neuropsychological outcomes and EOR were not associated with most of the clinical or radiological factors under analysis (**Table 1**). Pre-op seizure control, brain mapping strategy, and tumor relation to deep perforators were significantly associated to the EOR. To assess the predictive value of these variables on EOR, a backward stepwise binomial logistic regression was performed. The logistic regression model was statistically significant, c(3) = 20.236, p <.0001. The model explained 28% (Nagelkerke R) of the variance in EOR and correctly classified 78% of the cases. Specifically, all the three variables were statistically significant (pre-op seizure control, p = .006, Exp(B) = .216; relation to other deep perforators, p = .021, Exp(B) = 3.370; brain mapping, p = .012, Exp(B) = .249).

#### Brain Mapping Strategies

Brain mapping influenced post-operative outcomes (**Table 1**). The two groups previously defined based on the surgical strategy applied (patients treated before 2015 versus patients treated after) were comparable for patients’ and tumor characteristics (**Table 4**).

**Table 4:** Clinical, imaging, surgical, and postoperative neuropsychological features of patients operated before and after 2015.

Category	No. of patients, n (%)	Pre 2015	Post 2015	*p-value*
		28 (29.5)	67 (70.5)	
**Age, years**	Range	23.2–66.8	19–58.6	*0.594*
	Median	39.95	40.25	
	Mean	40.88	40.89	
**Sex, n (%)**	Males	20 (71.4)	39 (58.2)	*0.225*
	Females	8 (28.6)	28 (41.8)	
**Previous surgery, n (%)**	No	12 (42.9)	38 (56.7)	*0.263*
	Previous surgery	16 (57.1)	29 (43.3)	
**Clinical history, n (%)**	>6 months	16 (57.1)	27 (40.3)	*0.176*
	<6 months	12 (42.9)	40 (59.7)	
**Neurological deficit, n (%)**		8 (28.6)	20 (29.9)	*1.000*
	Language	6 (21.4)	10 (14.9)	
	Motor	1 (3.6)	10 (14.9)	
	Visual	1 (3.6)	2 (3)	
**Seizures, n (%)**		23 (82.1)	47 (70.1)	*0.309*
	Focal	28 (75)	46 (68.7)	
	Generalized	7 (25)	21 (31.3)	
**Seizures control, n (%)**	Control	13 (46.4)	30 (44.8)	*1.000*
	No	15 (53.6)	37 (55.2)	
**Side, n (%)**	Left	19 (67.9)	41 (61.2)	*0.643*
	Right	9 (32.1)	26 (38.8)	
**Location, n (%)**	Pure insular	4 (14.3)	10 (14.9)	*1.000*
	Opercular extension	24 (85.7)	57 (85.1)	
**MRI borders, n (%)**	Diffuse	16 (57.1)	44 (65.7)	*0.488*
	Compact	12 (42.9)	23 (34.3)	
**Pre-op volume in cm3**	Range	18.9 - 103.5	10.3 - 144.1	
	Mean	76.30	76.92	*0.921*
	Median	73.54	69.49	
**Anesthesia**	Awake	16 (57.2)	54 (80.5)	***0.019***
	Asleep	12 (42.8)	13 (19.5)	
**Brain mapping**	M & L	11 (68.8)	4 (7.4)	***0.000***
	M & L & C & H & V	5 (31.3)	50 (92.6)	
**EOR**	GTR	14 (50)	56 (83.6)	***0.002***
	Subtotal/Partial resection	14 (50)	11 (16.4)	
**New-onset neuropsychological deficit at 3 months**				
***Language***		5 (17.8)	11 (16.4)	*1.000*
***Memory***		1 (3.5)	6 (8.9)	*0.665*
*Non-dominant hemisphere*		0	0	
**Visuospatial abilities & praxis**		9 (32.1)	3 (4.5)	***0.001***
*Non-dominant hemisphere*		5 (83.3)	1 (16.7)	
***Attention & executive functions***		8 (28.6)	5 (7.5)	***0.005***
*Non-dominant hemisphere*		4 (66.7)	2 (33.3)	
***Fluid intelligence***		3 (10.7)	0 (0)	***0.030***

Clinical, imaging, and surgical features of patients categorized in two temporal series, pre- versus post-2015. The table also reports the numerosity and percentage of new-onset neuropsychological deficits (at least one pathological test score respect to pre-op) at 3 months evaluation for the patients under analysis submitted to surgery pre- and post-2015. The numerosity for patients with non-dominant lesions and new-onset neuropsychological deficits are illustrated for memory, visuospatial abilities & praxis, and attention & executive functions; the percentages are calculated on the sum of new-onset deficits in non-dominant lesions in the whole cohort (both pre- and post-2015 patients). In bold: P-values<0.05.

In the group of patients treated before 2015, the neuropsychological disturbances rate at 3 months was higher, particularly in visuospatial abilities and praxis (55.5%) and attentive and executive functions (44.5%) in non-dominant tumors, in which resection was performed asleep. Mean EOR was only 73.5%, a GTR was achieved in 50% of cases. Residual tumor remnants were most commonly cited in zone III, mainly in dominant tumors, due to difficult access to the insula posterior portion.

Among patients treated after 2015, the use of an extensive brain mapping decreased the rate of deficits in visuospatial abilities and praxis (4.5%), fluid intelligence (0%), and attentive and executive functions (7.5%). Furthermore, the EOR increased significantly; mean EOR was 98% and a GTR was achieved in 83.6%.

#### Dealing With Vascular Structures

Immediate postoperative MR documented DWI alterations in 19 cases (20%); most of these alterations were no more visible on MR study performed 3 months after surgery (**Table 1**). Out of these 19 cases, 11 patients developed postoperative neurological deficits concordant to ischemia location. In our series, the presence of ischemic insult detected on postoperative DWI associated with the persistence of new-onset postoperative neurological deficits at 1-month evaluation: eight out of the 11 patients with post-op ischemia and concordant new-onset neurological deficit maintained their deficit at 1-month evaluation, versus only 11 patients out of 33 with a new-onset deficit but no postoperative ischemia (p = .050). Furthermore, all three patients with language disturbances at 1 year presented postoperative DWI abnormalities along the course of eloquent tracts (2 along *Arcuate-Fasciculus*, 1 along *Inferior-Fronto-Occipital-Fasciculus*).

The location of LSA and its relation to tumor volume, reported in the literature as crucial (27, 28), were not predictive of postoperative ischemic insults. Tumor infiltration of other deep perforators, instead, was associated with a higher chance of postoperative ischemia in consonant areas (p = .01), persistence of new-onset motor deficits at 1-month (p = .05), and minor EOR (p = .05).

## Discussion

The present is the first study focusing exclusively on giant insular gliomas reporting results on EOR with full neurological, neuropsychological, and QoL outcomes. We showed that extensive resection is feasible and associated with mild long-term morbidity when a transcortical approach with extensive awake mapping is applied.

In giant insular tumors, growing data is currently available on the oncological effect of surgery; however, the assessment of the functional impact is limited to neurological examination only, and data on neuropsychological and QoL evaluations are scarce or lacking (**Table 5**) (4–6, 9–11, 29–32). The availability of this information is crucial to design and improve surgical approaches tailored to achieve the balance between extensive resection and full patient integrity. Resection of giant insular gliomas faces two major challenges that impact the likelihood of achieving a GTR and preserving patients’ integrity: dealing with the complex functional networks surrounding the insula and resecting the tumor from deep vascular structures (27, 28). Previous studies advocated the advantages of the transcortical approach for these purposes (1, 33). However, they evaluated surgical approaches and functional results of resection only in terms of the neurological examination. This novel report investigated surgical strategy, EOR, neurological, neuropsychological, and QoL outcomes in an extensive series of giant insular tumors.

**Table 5:** Clinical studies on giant insular gliomas resection.

Study details	N° of giant insular gliomas, n. (%)	Volume in cm^3^, median	LGG, n. (%); HGG, n. (%)	EOR >90%, n. (%)	Rate of permanent neurological deficits, n. (%)		NPS data, n. (%)	QoL data, n. (%)
					Language	Motor		
Sanai et al. (5)	14 (13.5)	n/a	8 (57.1); 6 (42.9)	24 (23.1)*	1 (0.9)*	3 (2.8)*	n/a	n/a
Skrap et al. (10)	46 (74)	108 (mean)*	53 (80.3); 13 (19.7)*	22 (33.3)*	2 (3)*	2 (3)*	n/a	n/a
Wu et al. (9)	n/a	43.1*	18 (55); 15 (45)*	median EOR 83.4%*	1 (3)*	2 (6)*	33 (100)	n/a
Martino et al. (6)	17 (77.2)	85.9 (mean)*	15 (68.2); 7 (31.8)*	EOR > 80% in 9 (52.9)	2 (9)*	0	n/a	n/a
Hervey-Jumper et al. (4)	12 (9.3)	91.2	70 (54.3); 59 (45.7)*	51 (39.5)*	1 (0.8)*	3 (2.4)*	n/a	n/a
Zhuang et al. (29)	3 (10)	80*	21 (70); 9 (30)*	23 (77)*	3 (11)*	2 (7.1)*	n/a	n/a
Eseonu et al. (11)	10 (13.5)	43.6*	25 (33.8); 49 (66.2)*	40 (54)*	0 (0)*	2 (2.7)*	n/a	n/a
Hameed et al. (30)	150 (58.8)	80.39	107 (71.3); 43 (28.7)	90 (60)	3 (1.74)	11 (6.40)	n/a	n/a
Mandonnet (31)	7 (58.3)	78	7 (100); 0	4 (57.1)	0	0	12 (100)	12 (100)
Li et al. (32)	29 (11.5)	106.2	149 (58.9); 104 (41.1)*	10 (34.5)	3 (1.2)*	5 (2)*	n/a	n/a
Rossi et al., 2020 (present study)	95 (100)	76.74	69 (73.7); 26 (26.3)	70 (73.7)	3 (3.2)	0	95 (100)	95 (100)

The numerosity of giant insular glioma cases with respect to total cases included in the study, preoperative volume in cm^3^ (median, except two cases), histological grade subdivision, rate of the extent of resection (EOR) >90%, rate of permanent postoperative neurological deficits, and presence of neuropsychological (NPS) and quality-of-life (QoL) data are reported. *Data presented refer to all cases under analysis in the study, not exclusively to giant insular glioma cases.

**Figure 2:**
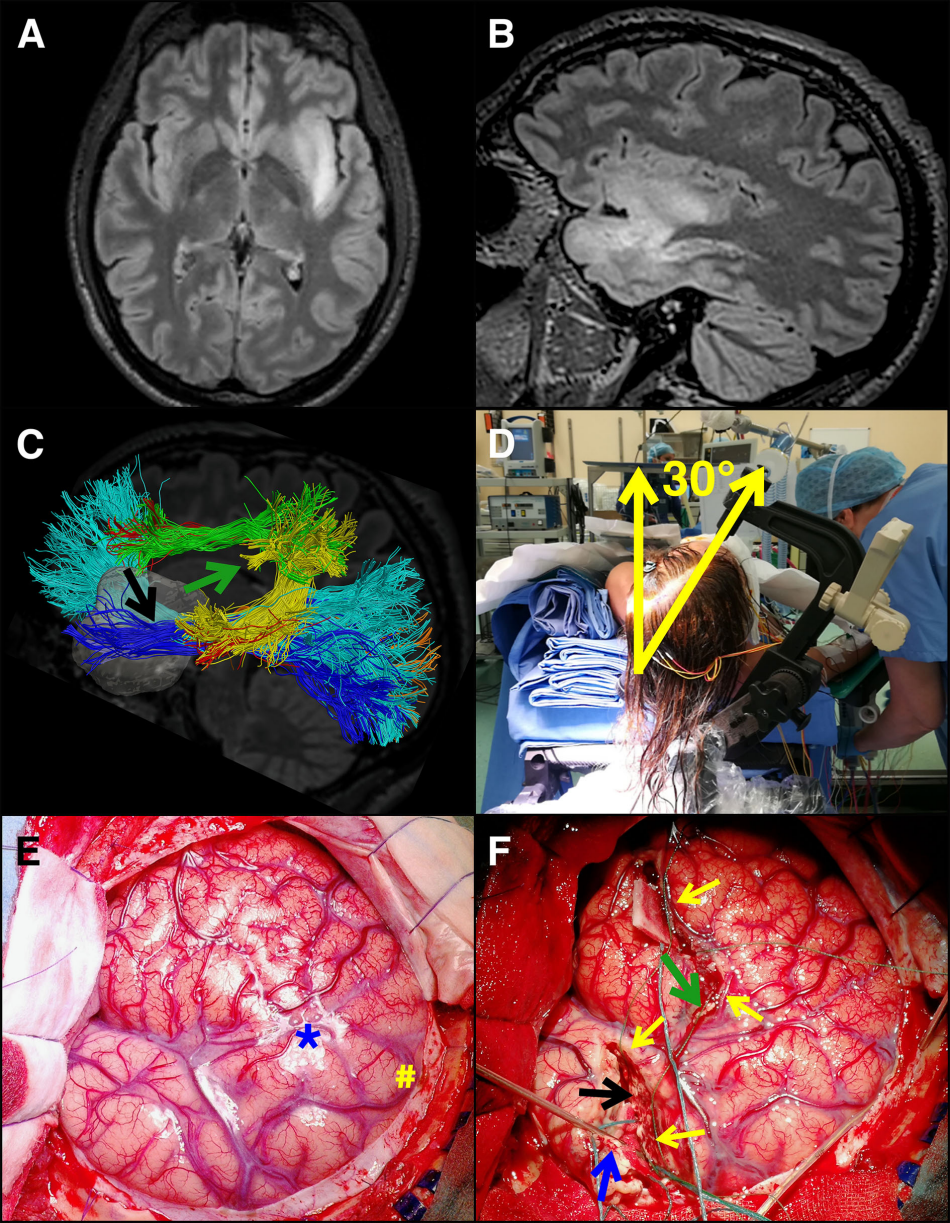
Resection of a giant insular glioma in the dominant hemisphere. A young patient presented with sensory-motor seizures. She was submitted to MR, which illustrated a presumptive giant insular low-grade glioma in the dominant hemisphere. **(A, B)** Pre-op axial and sagittal, respectively, FLAIR images. Tumor volume was 42.5 ml. The patient was submitted to surgery under asleep-awake-asleep anesthesia and extensive cortical and subcortical mapping (motor, language, cognitive, haptic, visual) and neurophysiological monitoring (EEG, ECoG, MEP, SSEP). **(C)** HARDI – DTI map of the main tracts surrounding the tumor (Inferior-Longitudinal-Fasciculus is in *blue*, optic radiation in *orange*, Inferior-Fronto-Occipital-Fasciculus in *cyan*, Arcuate-Fasciculus posterior segment in *yellow*, AF long segment in *red*, anterior segment [Superior-Longitudinal-Fasciculus III] in *green*). The two surgical corridors identified with the aid of brain mapping in the awake phase, also displayed in F, are highlighted by *arrows* (*green arrow*, frontal corridor consisting in SLF III and AF long segment above, and A474 F posterior segment posteriorly; *black arrow*, temporal corridor consisting in ILF inferiorly and posteriorly, IFOF and optic radiations superiorly and posteriorly). **(D)** intraoperative picture of the patient positioned supine with the shoulder elevated of 30° and the head tilted of 30° toward the tumor opposite side. **(E)** intraoperative picture of the large bone flap designed to expose the tumor area and the functional landmarks to be identified during the awake phase (M1 and vPM, in the picture marked with # and * respectively). **(F)** Intraoperative picture taken at the end of the awake phase, where the corticectomy on frontal and temporal opercula is visible, along with the posterior functional margin of the resection (secured by patties and indicated by *yellow arrows*). Subcortical identification of functional boundaries and functional disconnection of the frontal and temporal lobes and superior insula was performed by subcortical mapping; surgical patties were placed to indicate these boundaries (arrows). A monopolar probe was placed to indicate the temporal resection posterior border (indicated with a *blue arrow*). The use of extensive mapping allowed the identification of two surgical corridors, the first in the inferior and posterior margin of the frontal lobe (green arrow) and the second in the superior and posterior margin of the temporal lobe (black arrow). This approach, along with head positioning tilted of 30°, guaranteed optimal surgical access to the superior and inferior portions of zone II (frontal corridor, green arrow) and III (inferior corridor, black arrow), allowing to resect the tumor in these areas without damaging functional (visuospatial, cognitive and language sites). Final tumor removal was then performed under motor mapping (by HF) and continuous MEP-SEP recordings. Histo-molecular diagnosis revealed a grade II oligodendroglioma.

Our data are in line with the preferential use of a transcortical approach. Most of the cases under analysis presented tumor involvement of frontal and temporal opercula, target of resection. Dealing with giant insular tumors, the trans-Sylvian approach would have been inadequate to gain access to posterior insular zones (II and III). Furthermore, despite being less invasive, it presents the risk of damaging relevant vascular structures and secondary injury to frontal and temporal opercula by excessive or prolonged retraction. The transcortical approach adopted provided the opportunity to locate functional boundaries since the beginning of resection, obtaining a functional disconnection of the tumor from surrounding circuits in the frontal and temporal lobe. This helped with resection in Berger-Sanai zones I and IV; ensuring more extensive exposure of the insula also facilitated removal in Berger-Sanai zones II and III, the most challenging because of the heavy crossroads of functional networks, which limits surgical accessibility. By using this approach, mean EOR in our series was 92.3%, median 99%. EOR was associated with the type of mapping adopted and consequent surgical strategy. In the group of patients treated before 2015, mean EOR was 73.5%, and a GTR was achieved in half of the cases, while among patients treated after 2015, mean EOR was 98%, and a GTR was achieved in most of the cases. This cannot be explained simply by the learning curve of the senior surgeon; furthermore, the percentage of tumors in the dominant hemisphere in the groups before and after 2015 were comparable, as well as the use of intraoperative technologies adopted (neuro-navigation, ultrasound, intraoperative neurophysiological monitoring and mapping tools). Indeed, the use of extensive mapping, routinely adopted during the awake phase after 2015, inclusive of praxis, cognitive, and visual tasks, enabled the design of two large surgical corridors located, the first, in the inferior and posterior region of the frontal lobe, and the second, in the superior and posterior margin of the temporal lobe (**Figure 2**). This approach, along with head positioning tilted contralaterally of 30°, guaranteed optimal surgical access to insular zones II and III, while preserving long-term patient functional integrity as documented by the lower rate of post-op deficits in visuo-constructional abilities, fluid intelligence, and attentive & executive functions, among patients treated after 2015.

The same figures affected both neurological and neuropsychological outcomes. Functions declined in the immediate postoperative period and progressively recovered afterward, being language the most affected. The use of hMT mapping improved resection and dramatically decreased praxis abilities deficits (19). Similarly, the introduction of cognitive and visual mapping also in non-dominant sided tumors decreased cognitive (memory and attentive and executive function) and visual deficits (20).

Ischemic insult was the most relevant factor associated with short and long-term functional outcome: permanent deficits in language (3.2%) were associated with the onset of ischemic abnormalities in the white matter along the course of language tracts; postop DWI alterations were associated with the persistence of new-onset postoperative concordant neurological deficits at 1-month evaluation.

**Figure 3:**
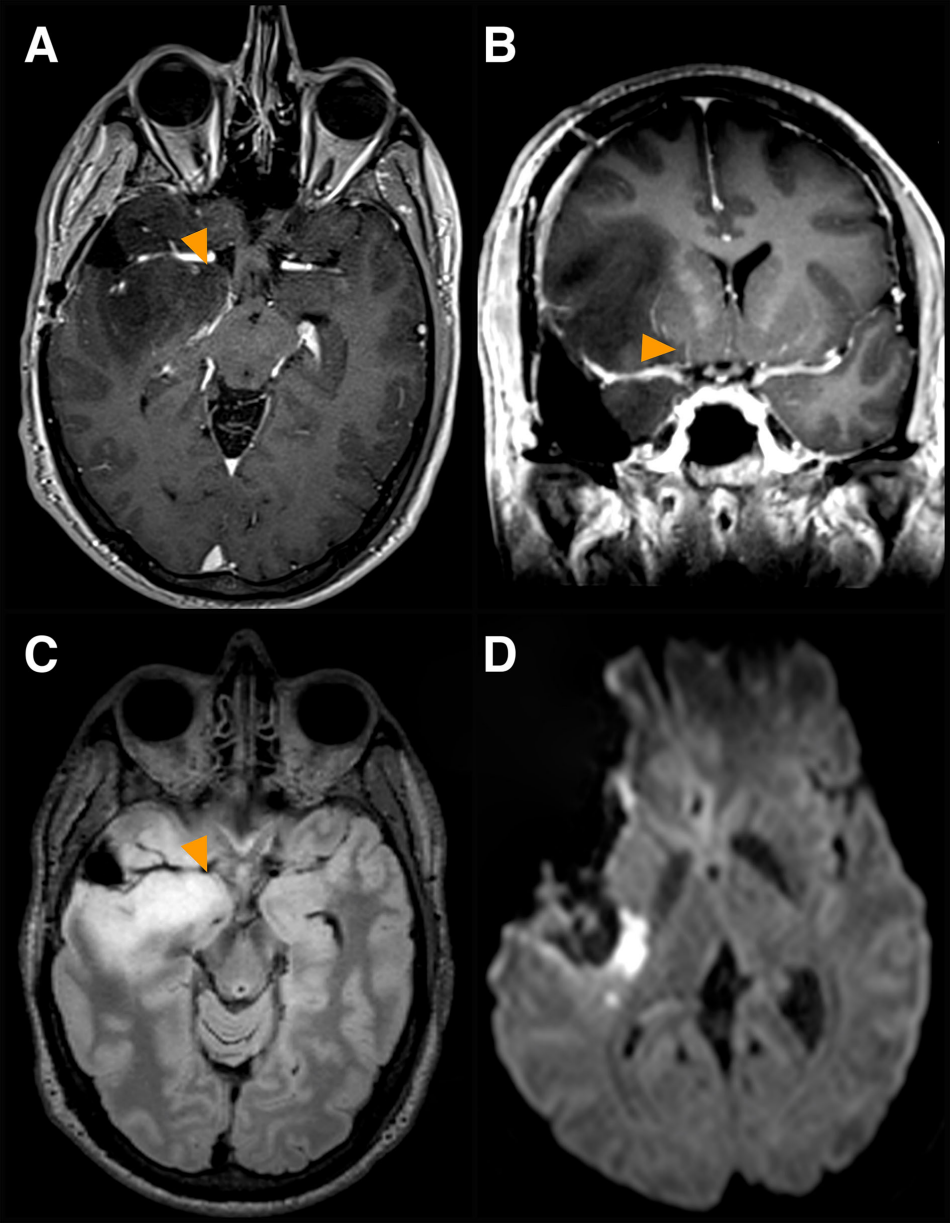
Involvement of other deep perforators. A case of a dominant fronto-tempo-insular low-grade glioma is presented. **(A, B)** Pre-op axial **(A)** and coronal **(B)** volumetric 3T MR T1 post-Gadolinium images displaying tumor involvement of other deep perforators (*yellow arrows*), arterial branches originating at the origin of the anterior choroidal artery from the internal carotid artery, medial to the LSA complex arteries. **(C)** corresponding pre-op axial FLAIR image. **(D)** post-op DW axial image, displaying ischemia posteriorly to the surgical cavity at the level of the posterior limb of the internal capsule. Severe MEP fluctuations and significant reduction occurred at the end of the resection. SEPs were unchanged. Arterial pressure was increased. The patient woke up with severe upper and lower limb paresis, which recovered progressively and entirely within 3 months after rehabilitation.

In all procedures, after functional disconnection of the tumor and surgical corridors delineation through frontal and temporal opercula, tumor removal from deep layers in proximity to the internal capsule and basal ganglia was performed under general anesthesia. Surgical maneuvers at this stage bring about the risk of ischemic insults. In our series, the tumor mass relation to other deep perforators, not to LSA complex arteries, was associated with a higher chance of postoperative ischemia in consonant areas. In the transopercular approach, early identification of the horizontal branch of the M1 tract of the MCA led to localization of the site of origin of LSA arteries, leaving these vessels always covered by an arachnoid layer; instead, other deep perforators always laid deep to the surgical plane (**Table 1**, **Figure 3**).

Furthermore, tumor infiltration of other deep perforators predicted a minor EOR. MEP changes anticipated the onset of motor decline during tumor dissection along deep perforators; resection was necessarily discontinued when fluctuations in MEP amplitude were detected. To note, most of MEP significant changes occurred suddenly, at the end of resection, highlighting the current difficulties in establishing a hierarchic series of predictive changes. However, the availability of such information enabled all measures (saline irrigation, arterial pressure increase) to be taken promptly to reduce the functional impact of ischemic events.

Between patients treated before 2015 and patients treated after, the approach to insular tumor resection was different. In the first group, dissection was performed from lateral to medial due to limited resection of the basal frontal lobe. In the second group, extensive cognitive, other than language, mapping in the frontal lobe, to secure access to zone I, led to more extensive exposure; this provided dissection of insular tumors in a rostrocaudal direction, from deep perforators to the internal capsule, working alongside these vessels and maintaining their pial plane in order to reduce the risk of inducing ischemic insults.

The high rate of functional preservation afforded by the combined use of the transcortical approach and extensive awake mapping was confirmed by QoL assessment. A considerable number of patients returned to work and everyday life, particularly among lower-grade glioma patients.

The main limitation of the present study is to be retrospective and single institution based. However, it is the only large study focusing solely on the giant subtype of insular gliomas, associating data of the surgical approach and EOR to those of neuropsychological and QoL evaluations. It stresses the role of advancement in intra-operative methodology to improve neurosurgical and functional results. It further supports the importance of neuropsychological and QoL evaluations for patients’ assessment and surgical strategy design.

A transcortical approach with extensive awake brain mapping enables giant insular gliomas resection extension preserving patients’ functional integrity in view of the presented data and experience. Perioperative ischemic insults represent the principal risk factor for long-term and permanent morbidity in this surgery. In this regard, the relation between tumor mass and deep perforators can predict perioperative ischemic insults.

## Data Availability Statement

The raw data supporting the conclusions of this article will be made available by the authors, without undue reservation.

## Ethics Statement

The studies involving human participants were reviewed and approved by IRB-1299-Humanitas-Research-Hospital. The patients/participants provided their written informed consent to participate in this study.

## Author Contributions

LG, MRo, LB: conception and design of the study. LB: directed and executed surgical procedures and intraoperative brain mapping. LG, MRo, MC, TS, GC, FA: acquired data and performed statistical analysis. LG, MRo, HH: produced figures. LB, LG, MRo, MRi: wrote the first version of the draft. LG, MRo, MC, TS, FA, AL, GP, HH, PZ, FV, GC, MRi, LB: discussed and interpreted the results. All authors contributed to the article and approved the submitted version.

## Funding

Sources of Support: AIRC (Associazione Italiana Ricerca sul Cancro), IG 18482, to LB.

## Conflict of Interest

The authors declare that the research was conducted in the absence of any commercial or financial relationships that could be construed as a potential conflict of interest.
